# Potential lead-free small band gap halide double perovskites Cs_2_CuMCl_6_ (M = Sb, Bi) for green technology

**DOI:** 10.1038/s41598-021-92443-1

**Published:** 2021-06-21

**Authors:** Muskan Nabi, Dinesh C. Gupta

**Affiliations:** Condensed Matter Theory Group, School of Studies in Physics, Jiwaji University, Gwalior, 474 011 India

**Keywords:** Energy science and technology, Materials science

## Abstract

Explorations of stable lead-free perovskites have currently achieved substantial interest to overcome the instability and avoid toxicity related issue faced with the lead-based perovskites. In this study, we have comprehensively studied the stability, nature and origin of electronic, transport and optical properties of inorganic halide double perovskites, which could provide a better understanding of their possible potential applications. The density functional theory is used to investigate the different physical properties of these materials. The stability of these cubic materials is validated by optimizing the structure, tolerance factor, mechanical stability test. The materials are small band gap semiconductors with outshining optoelectronic performance. Due to high optical absorption, high conductivity and low reflectivity they have great potential to be used for optoelectronic application purpose. Because of small band gap we have also investigated the variation of various transport parameters with chemical potential. The semiconducting nature of materials results in ZT close to unity predicting its excellent application in thermoelectric technology.

## Introduction

Due to increasing energy demands, there has been an increase in the consumption of fossil fuels which sufficiently contributes to the pollution levels and cause global warming. So, an eco-friendly power source is needed to meet the energy crises. To over-come this issue, solar cells made up of silicon proved to be an ideal solution. However, these silicon-based solar cells not only have complicated production procedure but also have less power conversion efficiency. So, there is a sustained research interest toward alternative photovoltaic (PV) materials produced with cost-competitive, facile, and environmentally friendly technologies. In this field perovskite solar cells have gained much progress during the last few decades increasing the efficiency from 3.8% in 2009 to 22.7% in 2017 at the lab-scale^1–5^. Hybrid halide perovskites with a general formula ABX_3_ where A is a monovalent cation such as methylammonium (MA^+^), formamidinium (FA^+^), and B is a divalent cation such as Pb^2+^ and X are halides such as Cl, Br, I are the most commonly studied materials for optoelectronic applications. But there are many issues associated with the commercialization of lead based solar cells particularly instability and toxicity^6,7^. Owing the thermodynamic and environmental stability Cesium halide perovskites proved to be saviors to overcome the less-stability issue of organic cations such MA^+^, FA^+^ against the environmental condition. Since lead is a very toxic heavy element has hazardous effects not only on the environment but also on human beings. Therefore, it is very important to reduce or eliminate lead from PV devices^8,9^. So, keeping this in view research is going on to replace the lead from perovskites by some other non-toxic elements. The replacement for lead must fulfill certain criteria in order to match the excellent performance of the lead-based perovskites. The alternative to lead must be low-cost, easily recycled, should exhibit excellent optoelectronic properties. In addition to being competitive with currently established PV technologies, they should also satisfy some commercial necessities like flexibility, long-term stability, and scalability^10,11^. The issue is being addressed by the replacement of lead-based perovskites with environment friendly lead-free halide-based perovskites. The success arises because these semi-conducting halide perovskites possess outstanding optoelectronic properties such as high optical absorption coefficient, bandgap that can be tuned, long carrier recombination lifetimes, high carrier mobility, small electron/hole effective masses, and high molar extinction coefficient. Among double halide perovskites, Bi and Sb-based families have drawn a remarkable interest. Cs_2_MBiCl_6_ (M = Ag, Cu, Na) and Cs_2_MBiCl_6_ (M = K, Rb and Cs), Cs_2_NaBX_6_ (B = Sb, Bi; X = Cl, Br, I) have shown excellent stability and good optoelectronic application with smaller and larger band gaps correspondingly^12,13^. The nanocrystals of Cs_2_CuMCl_6_ (M = Bi, Sb) as well as other related family members have been experimentally reported^14–21^.

Besides the PV, thermoelectric technology has also achieved significant interest during last few decades. Thermoelectric efficiency of materials is given by dimensionless figure of merit (ZT) given by; $ZT = \frac{{S^{2} \sigma T}}{{\kappa _{e}  + \kappa _{l} }}$, where S is Seebeck coefficient, σ is electrical conductivity, T is absolute temperature, κ_e_ is electronic thermal conductivity and κ_l_ is lattice thermal conductivity^22,23^. A material with ZT ~ 1.0 is considered a good thermoelectric material^24^. Such a high ZT value may be obtained when the power factor (PF) is high and thermal conductivity is low. Despite ultra-low thermal conductivity arising because of occupation of cations in the octahedral structure along with high charge mobility, it is quite surprising that these halide double perovskites have been mostly studied for optoelectronic applications. There are only small experimental studies conducted to study thermoelectric efficiency, but the interest towards the thermoelectric response of halide perovskites is now increasing. Theoretical calculations have claimed that the halide and hybrid perovskites could achieve ZT value equal to one. A variety of perovskites have attained figure of merit equal to one like in SrTiO_3_ (La substituted)^25^, MASnI_3_ (ZT ~ 1.0)^26^, CsSnCl_3_ (ZT ~ 1.0)^27^, Cs_2_AgBiX_6_ (X = Cl, Br) (ZT ~ 1.0)^28^. Fatima Aslam et al.^29^ studied Cs_2_InAgX_6_ (X = Cl, Br, I) suggesting that these materials are exhibiting tunable direct energy band gaps that can be employed in practical devices for energy harvesting applications. Motivated by their small band gap and unmatchable desirable properties, we have tried to explore these two materials for optoelectronic application and extended our study to unravel their thermoelectric properties which they equally justify.

### Computational methods

The first principle method with the help of *Wien2k* simulation code^30^ is used to calculate the electronic structure, optical and transport properties of the materials. The ground state properties are calculated by solving Kohn–Sham equation properly. For the said purpose, different approximation methods like generalized gradient approximation (GGA), onsite coulomb interaction (GGA + U), modified Becke-Johson (mBJ) are utilized to approximate the only unknown term exchange–correlation potential in the state-of-art formulism^31,32^. Besides these methods we have also considered spin orbit coupling effect for the present set of materials. The unit cell volume is divided into muffin tin spheres where wave function shows atomic like character and interstitial space wherein plane wave basis set is employed. The extension of the basis set is controlled via R_MT_ *k*_max_ = 7 and *l*_max_ = 10 conditions, where R_MT_ is smallest muffin tin radii and *k*_max_ represent maximum value of *k*. To obtain the convergence of results the unit cell in the *k*-space is divided into a dense mesh of 1000-*k* points for integration over the Brillouin zone. As the thermoelectric parameters are sensitive to *k* point sampling therefore a high dense of 150,000 k points is utilized to calculate the same. The iterations for charge convergence between successive cycles converge up to 0.0001 e and energy up to 0.0001 Ry to obtain better results. The thermoelectric properties are determined under the approximation of constant relaxation time (τ) with the help of BoltzTraP code^33^. With the help of Gibbs2 code^34^ we have evaluated some of the thermodynamic parameters like Debye temperature and Grüneisen parameter.

### Structural properties

**Figure 1:**
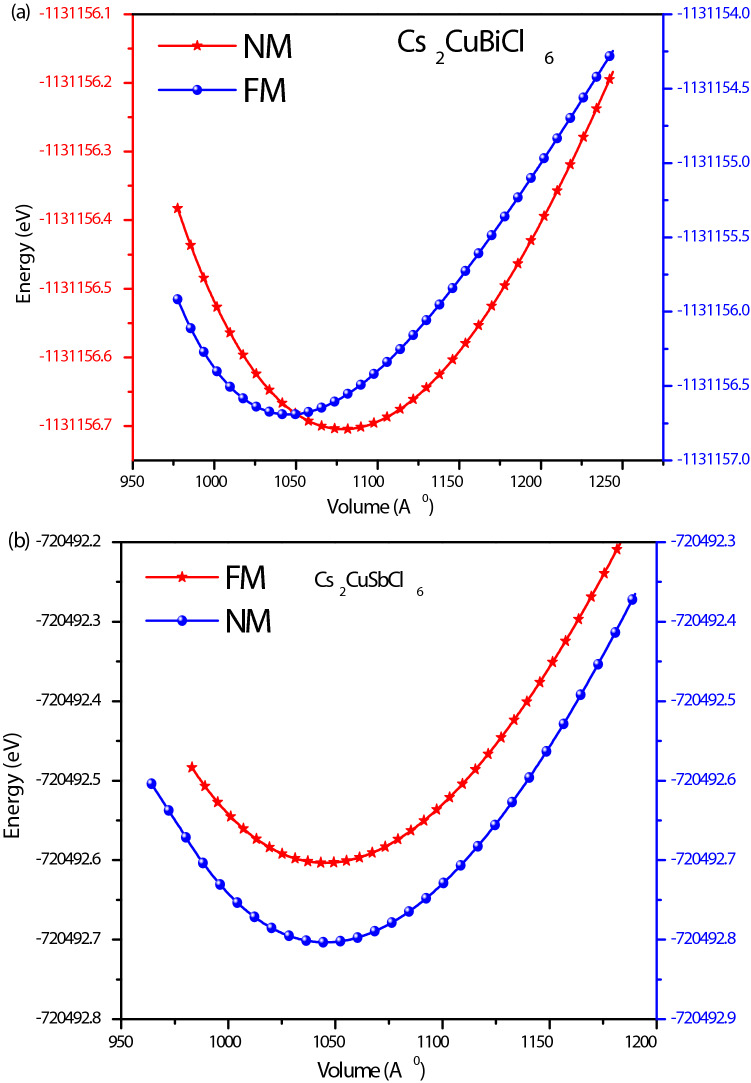
(**a**,**b**) Energy versus volume optimization curve of Cs_2_CuMCl_6_ (M = Sb, Bi) in both spin-polarized and non-polarized states.

The structural stability of the materials can be determined by various factors like optimizing crystal structure by utilizing the Birch Murnaghan equation of state^35^, Goldschmidt’s rule from the effective ionic radii or bond length^36^. The structural stability, the band structure and carrier transport performance of materials and their specific application to a large extent is predominately determined by the combination of cations and anions. The band profile of halide double perovskites with general formula A_2_B(I)B′(III)X_6_ are predominantly decided by B (I)-, B(III) and X-site atoms. Fig. S1a (Supplementary Information) shows a possible combination of different cations from the periodic table for the possible formation of halide double perovskites^37^. The correct combination of these cations leads to the excellent properties of these materials. In the present case, the structural optimization of the titled halide double in ferromagnetic (FM) and non-magnetic (NM) perovskites is done by utilizing the Birch-Murnaghan equation of state which justifies the cubic stability with the non-magnetic (NM) ground state as the stable state as shown in Fig. 1a,b. Also, the cubic stability is determined from the tolerance factor. The ground state parameters of these materials are quoted in Table 1 which agrees well with the previously reported results^14,15^. The pictorial representation of the titled perovskite in which cesium is enclosed by a cage of 12 halide atoms (Cl) while as *d* block element Cu and *p* group elements (Sb/Bi) lie in octahedral of halide atoms having coordination 6 to these atoms is shown in Fig. S1b (Supplementary Information).

**Table 1:** The optimized lattice parameters of cubic Cs_2_CuMCl_6_ (M = Sb, Bi) with space group $Fm\overline{3} m$ in both spin-polarized and non-polarized states.

Compound	State	a (Å)	Previous reported	V (Å)^3^	B (GPa)	B′	E_0_ (eV)
Cs_2_CuSbCl_6_	FM	10.53	10.52^14^, 10.52^15^	1045.31	32.70	5.06	− 720,492.60
	NM	10.53		1045.33	32.67	5.02	− 720,492.80
Cs_2_CuBiCl_6_	FM	10.64	10.61^14^ 10.33^16^	1079. 05	31.72	4.84	− 1,131,156.69
	NM	10.64		1078.93	31.62	5.09	− 1,131,156.70
Cs_2_AgBiCl_6_	–	10.70^28^	10.77	–	29.98	6.37	–
Cs_2_AgSbCl_6_	–	10.70^14^	10.70	1225.36	–	–	–

### Second-order elastic constants and mechanical stability

The elastic constants and thereby mechanical behavior of these considered double halide perovskites are predicted with the help of the *Cubic Elastic package*^38^. Equilibrium cubic structure is deformed by applying small strains to predict second-order elastic constants. The cubic structure would be mechanically stable only if the deformed structures are at higher energy compared to the cubic phase. This leads to a limiting condition $C_{{11}}  - C_{{12}}  > 0,C_{{11}}  > 0,C_{{44}}  > 0,C_{{11}}  + 2C_{{12}}  > 0,C_{{12}}  < B < C_{{11}}$^39^ for elastic constants to be followed where C_11_ longitudinal elastic constant indicates elasticity along the axis of unit cell and C_12_ and C_44_ are shear elastic constants define elasticity in shape.

The elastic constants help to predict the response of any material to applied stresses. The second-order elastic constants (SOECs) in the present work along with the already reported values have been evaluated utilizing the energy-strain approach in the framework of GGA-PBE given in Table 2. All three elastic constants are non-negative and follow the Born stability criteria condition^40^. Thereby, advocate the mechanical stability of the materials. From the SOECs, the universal anisotropic factor (A^U^)^41^ is deduced. The deviation of A^U^ from unity signifies titled halide double perovskites are highly anisotropic. The anisotropy mainly originates because of the large difference in longitudinal and shear elastic constants. Using SOEC we have estimated mechanical parameters like Young’s (Y), shear (G), and bulk moduli (B), Poisson’s ratio (σ) using the mathematical relations reported elsewhere^42^. Further, by employing Elate: Elastic tensor analyzer^43^, we have analyzed the angular dependence of elastic modulus. The results reflect that Young’s modulus and shear modulus are highly anisotropic shown in Fig. S2a,b, while bulk modulus is isotropic as happens in cubic crystals. In addition to 3D graphical representation of directional elastic properties a quantitative analysis by reporting the minimal and maximal values of each modulus is reported in Table S1. Moreover, by executing the Reuss-Vogoit-Hill scheme^44^ we have defined the average values of different elastic moduli. The obtained results are summarized in Table S2. The C_11_-values for both double perovskites are greater than the other two shear elastic constants (C_12_ and C_44_) and also B being greater than G reflects that these materials show more resistance for volumetric deformation compared to the shape deformation. The Pugh’s ratio (B/G), Poisson’s ratio (σ), and Cauchy pressure (C_P_ = C_12_ – C_44_)^45–47^ are greater than their index values of 1.75, 0.26, and 0, respectively as can be seen from Table 2. These values thus signify Cs_2_CuSbCl_6_ and Cs_2_CuBiCl_6_ double perovskites are ductile. So, these materials can be used to design tools of varying shapes.

**Table 2:** Second-order elastic constants (SOECs) obtained by utilizing the energy-strain approach in the framework of GGA-PBE for Cs_2_CuMCl_6_ (M = Sb, Bi).

Parameters	Cs_2_CuSbCl_6_	Cs_2_CuBiCl_6_	Cs_2_AgBiCl_6_^28^
**Elastic constants**			
C_11_ (GPa)	57.21	56.27	50.03
C_12_ (GPa)	19.56	19.23	19.96
C_44_ (GPa)	5.05	4.05	7.76
Bulk modulus (B in GPa)	32.11	31.57	29.98
Shear modulus (G in GPa)	12.36	7.86	10.14
Pugh’s ratio (B/G)	2.59	4.01	2.96
Young’s modulus (Y)	32.86	21.77	27.35
Poisson’s ratio (σ)	0.32	0.38	0.35
Zener anisotropy factor (A^U^)	0.26	0.21	–
Cauchy pressure	14.51	15.18	–
Compression velocity (V_l_ in m/s)	3410.00	3180.00	3175
Shear sound velocity (Vs in m/s)	1520.00	1370.00	1534
Mean sound velocity (V_D_ in m/s)	3331.02	2971.98	1725
Debye temperature (θ_D_ in K)	151.17	133.38	164

Additionally, we have simplified the ultrasonic wave velocities of the titled double perovskites using SOECs and the density of the materials^48^. In cubic structure pure longitudinal (V_*l*_) and two transverse (V_T1_ and V_T2_) modes only happen along [100], [110] and, [111] direction. The magnitude of the sound wave velocity is obtained through the following equation, $V = \sqrt {\frac{{C_{{eff}} }}{\rho }}$^49^ where, the *C*_*eff*_ for different modes along different directions are defined in Table S3**.** These wave velocities in turn are used to estimate the average Debye velocity (mean sound velocity V_D_) using relation $V_{D}  = \left\{ {\frac{1}{3}\left( {\frac{1}{{V_{L} }} + \frac{1}{{V_{{T1}} }} + \frac{1}{{V_{{T2}} }}} \right)} \right\}^{{ - \frac{1}{3}}}$^50^. The calculated values of Debye velocity or mean sound velocity are presented in Table 2. Moreover, we have tallied the Debye temperature (θ_D_) of the Cs_2_CuSbCl_6_ and Cs_2_CuBiCl_6_ perovskites by using the Debye average velocity V_D_ in with equation, $\theta _{D}  = \frac{h}{k}\left\{ {\frac{{3n}}{{4\pi }}\frac{{NA\rho }}{M}} \right\}^{{\frac{1}{2}}} V_{D}$^51^. The obtained values of ultrasonic sound velocities and Debye temperature furthermore authenticate the anisotropic nature of the materials. Moreover, the high value of Debye temperature signifies these materials are stable at extreme temperatures and could be used for the fabrication of the devices.

### Electronic properties

**Figure 2:**
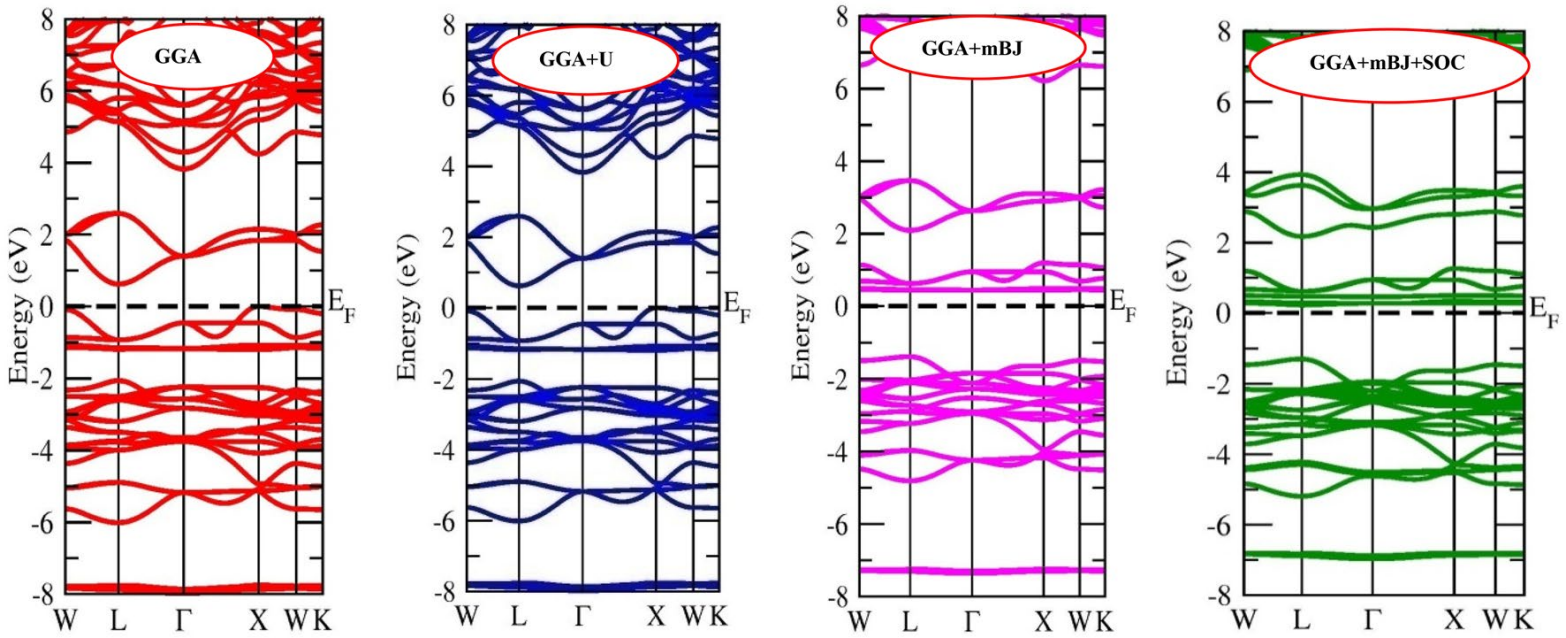
Band structure of Cs_2_CuSbCl_6_ calculated by GGA, GGA + U and GGA + mBJ and GGA + mBJ + SOC methods.

**Figure 3:**
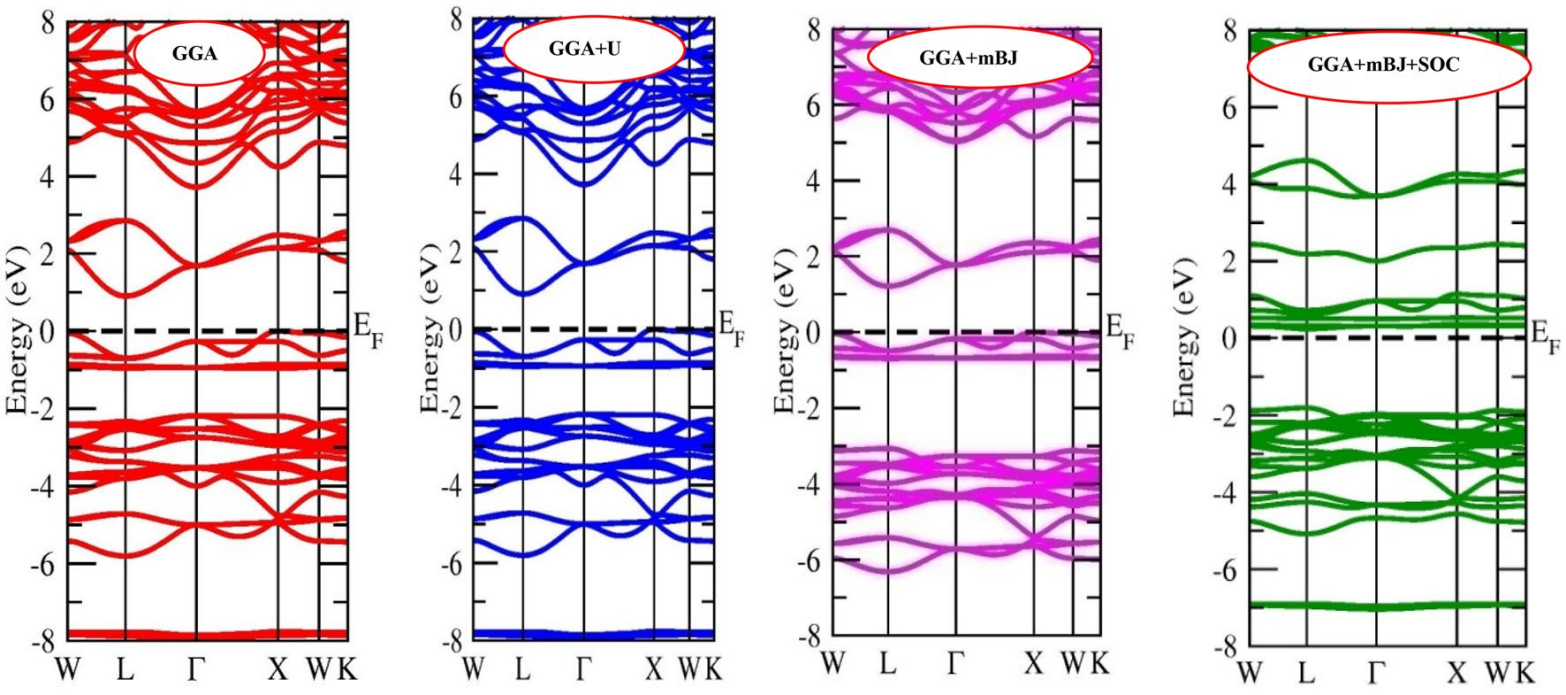
Band structure of Cs_2_CuBiCl_6_ calculated by GGA, GGA + U and GGA + mBJ and GGA + mBJ + SOC methods.

The applications of any material are profoundly characterized by the electronic properties which include band structure and distribution of electrons in these bands^52^. Herein, with the assistance of density functional theory, we have evaluated the electronic properties of inorganic halide double perovskites. The band structure calculated via non-spin polarized calculations on employing different approximation methods are provided in Figs. 2 and 3. It is clear that the Fermi level is unoccupied indicating the semiconducting nature for Cs_2_CuMCl_6_ (M = Sb, Bi). Also, the valence band maxima (VBM) and conduction band minima (CBM) are located at different symmetric points resulting in indirect band gap. The band gap calculated for Cs_2_CuSbCl_6_ and Cs_2_CuBiCl_6_ by GGA are ∼ 0.61 eV and ∼ 0.89 eV for Sb and Bi-based perovskites, respectively. On adding Hubbard potential and spin orbit coupling (SOC) potential to GGA, almost no change in the band gap is observed. However, on assisting mBJ potential to GGA, indirect band gaps increase to ∼ 1.00 eV for Cs_2_CuSbCl_6_ and ∼ 1.20 eV for Cs_2_CuBiCl_6_. The energy bands mostly populated of Cl-*p* and Cu-*d* stated are pushed away from Fermi level by mBJ-potential result in broadening of band gap. Since the mBJ potential effectively enhance the band gap, so we have further added the SOC and mBJ potentials altogether to GGA. By GGA + mBJ + SOC the band gaps turn out to be ~ 1.50 eV and ~ 2.0 eV for Sb and Bi-based materials, respectively, consistent with the experimental reported results. The mBJ + SOC effect removes the degeneracy of some states, thereby magnify the bandgap. The bandgap values obtained through different approximation in comparison with the experimental and theoretical reported values are summarized in the Table 3. The valence band (VB) is populated by the filled states, empty states enter to conduction band (CB), and partially filled states crossover Fermi level. The oxidation state and the number of remaining valence electrons in the respective oxidation states pre-determines which states would enter to the valence band or conduction band or occupy the Fermi level. In the entitled double perovskites, Cs and Cu have oxidation state + 1, Cl has − 1 oxidation state while the main block elements Sb and Bi are in + 3 oxidation state. In Cs_2_^+1^Cu^+1^ M^+3^Cl_6_^–1^(M = Sb, Bi) oxidation configuration charge of constituents is balanced; moreover, the constituents have only paired electrons.

**Table 3:** Calculated band gap and nature of band gap of Cs_2_CuMCl_6_ (M = Sb, Bi) in present study in comparison with experimental and other theoretical results available in literature.

Material	GGA	DFT method				Experimental value	Nature of bandgap
		GGA + U	GGA + SOC	mBJ	GGA + mBJ + SOC		
Cs_2_CuSbCl_6_	0.61	0.61	0.59	1.00	1.55	1.66^14^	Indirect
Cs_2_CuBiCl_6_	0.89	0.89	0.85	1.20	2.00	–	Indirect
		0.83^16^, 0.93^18^, 2.00^20^			–		
Cs_2_CuBiBr_6_		0.51^16^			–	–	
Cs_2_AgSbCl_6_		1.41^53^			2.41^53^	2.60^53^	Indirect
Cs_2_AgBiCl_6_		1.66^54^, 2.1^55^			2.60^54^, 2.62^55^	2.77^54^, 2.62^55^	Indirect
Cs_2_AgSbBr_6_		1.46^56^			1.46^56^	1.64^56^	–
Cs_2_AgBiBr_6_		1.7^55^			2.19^55^, 1.8^57^	2.06^55^, 2.3^57^	Indirect
Cs_2_InBiCl_6_		0.88^58^			0.92^58^	–	Indirect
Cs_2_AgTlCl_6_		0.00^59^			1.87^59^	1.96^59^	Direct
Cs_2_AgTlBr_6_		0.0^59^			0.63^59^	0.95^59^	Direct
Cs_2_LiGaBr_6_		0.73^60^			1.96^60^	–	Direct
Cs_2_NaGaBr_6_		0.45^60^			1.76^60^	–	Direct
Cs_2_AgInCl_6_		3.23^61^			–	3.33^61^	Indirect
Cs_2_AgBiI_6_		0.77^62^			0.89^62^	1.75^7^	Indirect

**Figure 4:**
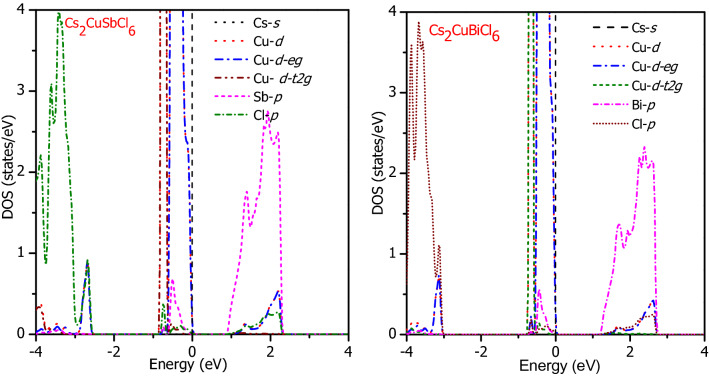
Partial density of states of Cs_2_CuMCl_6_ (M = Sb, Bi) calculated by GGA + mBJ scheme.

The qualitative description of the valence band and conduction band and the energy states associated with them is illustrated with the help of the density of states. The valence state Cs-*s*, Cu-*d*, Sb/Bi-*p* and Cl-*p* contribution toward the band composition obtained by GGA + mBJ is presented in Fig. 4. Among all the states, most interested states are *d*-states of Cu which are in the vicinity of Fermi level. The *p*-states of Cl gets electrons from cations mostly compose the VB. The Cs-*s* and Sb/Bi-*p* states are nowhere in the vicinity of the Fermi level; therefore, these states play a passive role in characterizing the electronic properties in these perovskites. The Cu-*d*-states in the octahedral field split into triplet *d-t*_*2g*_ and doublet *d-e*_*g*_ states, *d-t*_*2g*_ states being at lower energy. The *d-t*_*2g*_ can intake a maximum of six electrons (3↑ and 3↓) while as *d*-*e*_*g*_ state is filled by 4-electrons (2↑ and 2↓). Therefore, the electron filling in *d*-orbitals of Cu^1+^ is 3*t*_*2g*_ (↑), 3*t*_*2g*_ (↓), 2*e*_*g*_ (↑), and *2e*_*g*_ (↓). All the *d*-orbitals are filled for both spin channels therefore form the VB. Moreover, the crystal field splitting energy for the configuration is zero. The *p*-states of Cl gets electrons from cations are filled and happen to be in VB. Therefore, in Cs_2_CuMCl_6_ (M = Sb, Bi) the distribution of energy states is; Cu-*d* and Cl-*p* are completely filled lie in valence band below Fermi level and while as Sb/Bi-*p* and Cs-*s* are empty in the conduction band a small band at Fermi level. The constituent atoms have no unpaired electrons resulting in the non-magnetic character of these materials which is also confirmed from the structural optimization. The obtained semi-conducting nature along with small band gap values signifies that they can outshine in optoelectronic and thermoelectric applications. The partial densities of states obtained by GGA + mBJ + SOC are plotted in Fig. S3 and discussed in the Supplementary Information.

### Thermophysical properties

The variation of transport parameters like carrier concentration, Seebeck coefficient, conductivity, etc. with chemical potential and temperature is remarkable. So, to illustrate the chemical potential dependence of transport coefficients at different temperatures we have used constant relaxation time approximation under BoltzTraP code^33^. The magnitude of thermoelectrical parameters in semiconductors is mainly characterized by band structure, as the central contribution is from band gap, carrier type, carrier concentration, and carrier effective mass^63^. The transport behavior is directly linked with the energy bands within the Fermi level. In the Electronic Properties Section, it is found that by incorporating mBJ and mBJ + SOC potentials to GGA, the bandgap changes effectively. The bandgap predominately decides which carries (electrons and holes) take part in the transport phenomenon. So, the transport properties of both conductivities and Seebeck coefficient extensively depend on the value of the bandgap. The high Seebeck coefficient is shown by the insulators, while metals have the least Seebeck coefficient^64^. So, the narrower the band gap lesser would be the Seebeck coefficient and vice-versa. Conductivity on the other side is proportional to the effective charge carries. In Cs_2_CuMCl_6_ (M = Sb, Bi) perovskites, GGA + mBJ and GGA + mBJ + SOC sophistically improves the bandgap. So, to understand the role of bandgap underestimation on the transport properties, we have computed the transport properties by both approximations. The SOC results are presented in the Supplementary Information.

The variation of carrier concentration with chemical potential at different temperatures is presented in Fig. S4. The carrier concentration graph gives us an idea about the nature of transport carriers which describes electronic properties which in turn affects other transport parameters. The sign of the carrier concentration designates the nature of charge carriers; the negative sign indicates that electrons are majority carriers while as positive carrier concentration means holes are majority carriers^65^. The sharp variation in carrier concentration corresponds to the presence of bandgap/pseudo-gap in the band structure. As the temperature increase electron gain more thermal energy hence carrier concentration increases with an increase in temperature.

**Figure 5:**
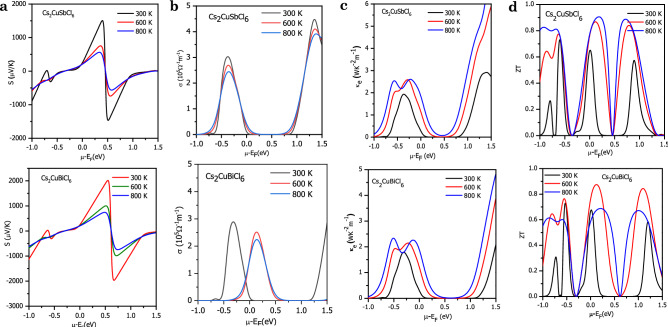
Variation in transport parameters with chemical potentials at different temperatures viz. (**a**) Seebeck coefficient; (**b**) Electrical conductivity; (**c**) thermal conductivity; (**d**) figure of merit (ZT). Different colors are used to distinguish the temperature: Black-300 K; Red-600 K; and Blue-900 K.

The Seebeck coefficient (S) as a function of chemical potential (μ-E_F_) at different temperatures (300 K, 600 K, 800 K) is plotted in Fig. 5a. For the entire region of chemical potential, the Seebeck coefficient displays prominent peaks and valleys. There are high-intensity peaks for the positive potential as well as negative potential at 300 K and these values decreases as temperature rise to 600 K and 800 K. The decreasing character is because bound electrons get excited by acquiring thermal energy, generate electron–hole pairs. The most prominent peaks are in the range of 0 to 1 eV as the bands are less dispersive with the forbidden region around Fermi level, thereby fewer charge carriers are around in this range. The maximum value of S is 2000 μV/K for Cs_2_CuSbCl_6_ and 1500 μV/K for Cs_2_CuBiCl_6_ at 300 K, respectively. On comparing the results of both these materials it can conclude that Cs_2_CuBiCl_6_ shows a better Seebeck coefficient because of the presence of a larger bandgap. The obtained results are in decent agreement with the already reported theoretical results of similar other materials, thereby validate our results^28,29,36^. The magnitude thermopower |S| obtained by GGA + mBJ + SOC illustrated in Fig. S5a is higher than the GGA + mBJ counter-partner; it is because the bandgap significantly increases with the incorporation of SOC.

The graphical variation of electronic conductivity (σ) as a function of chemical potential at temperature range 300 K, 600 K, and 800 K is demonstrated in Figs. 5b and S5b calculated by GGA + mBJ and GGA + mBJ + SOC, respectively**.** Because of the absence of energy bands around the Fermi level making the area desolate of charge carriers and hence the conductivity vanishes around μ-E_F_ = 0. But below or above the Fermi level the conductivity increases because of the presence of energy states. With the rise in temperature the conductivity in the vicinity of the Fermi level increases, because of band smearing. Certain energy states that were filled at T = 0 K becomes empty because with rise in temperature electrons make the transition from the valence band to the conduction band.

The total lattice thermal conductivity comprises of lattice part arising due to lattice vibrations and electronic part arising due to charge carriers. Here, we tried to evaluate both the components of total thermal conductivity with different chemical potentials at a temperature range 300 K, 600 K and 800 K as shown in Fig. 5c. As, thermal conductivity and electronic conductivity both depend on carrier concentration so, with change in chemical potential, they follow a similar profile of variation. However, the thermal conductivity increases abruptly with temperature compared to electrical conductivity. The results depicted by these agree well with the Wiedemann Franz law which states the proportional relation between them as follows: κ = σLT^66^. The lattice part of thermal conductivity is calculated with the help of Slack’s equation, $\kappa _{l}  = \frac{{A\theta _{D}^{3} V^{{\frac{1}{3}}} m}}{{^{{\gamma ^{2} }} \overline{N} ^{{\frac{2}{3}}} T}}$^22^. The equation suggests that κ_*l*_ is dependent on Debye temperature (θ_D_), volume (V), average molar mass per atom (m), Grüneisen parameter (γ), temperature (T) and number of atoms per unit cell ($\overline{N}$).The value of A is calculated as, $A = \frac{{2.43 \times 10^{8} }}{{1 - \frac{{0.514}}{\gamma } + \frac{{0.228}}{{\gamma ^{2} }}}}$^67^. The variation of these interdependent quantities such as Grüneisen parameter and Debye temperature with temperature has been plotted in Fig. S6a,b. Debye temperature is an important parameter that characterizes the thermal vibrations in a solid. It is the maximum temperature above which a solid behaves classically and the constituents exhibit coupled vibrations. The degree or extent of anharmonicity in a crystal is determined by Grüneisen parameter. As the temperature is increased the atoms start vibrating more rigorously which leads to an increase in anharmonic effects. From the plots, we can see that θ_D_ decreases while as γ increases with an increase in temperature. Finally, with the help of these interdependent quantities, we can evaluate the lattice thermal conductivity using Slack’s equation as shown in Fig. S6c. It can see that these thermodynamic parameters don’t change much with temperature so it is clear that κ_*l*_ depends mostly on the number of atoms ($\overline{N}$).

The most important parameter which scrutinizes the efficiency of thermoelectric materials is the dimensionless figure of merit^68^. The relation of ZT clearly signifies that it increases with electrical conductivity and Seebeck coefficient while it decreases with increasing thermal conductivity. Figures 5d and S5c (with SOC) display the figure of merit (ZT) values of Cs_2_CuSbCl_6_ and Cs_2_CuBiCl_6_ as a function of chemical potential at temperature 300 K, 600 K and 800 K. The obtained values of ZT are compared with reported results in the literature, tabulated in Table S4. Both these halide perovskites have prominent peaks with the highest peak having ZT nearly equal to 1. The high value of ZT can be attributed to the semiconducting nature of these materials. We can see from the graph that as temperature increases the magnitude of ZT begins to increase and reaches the value of at high temperature**.**

**Figure 6:**
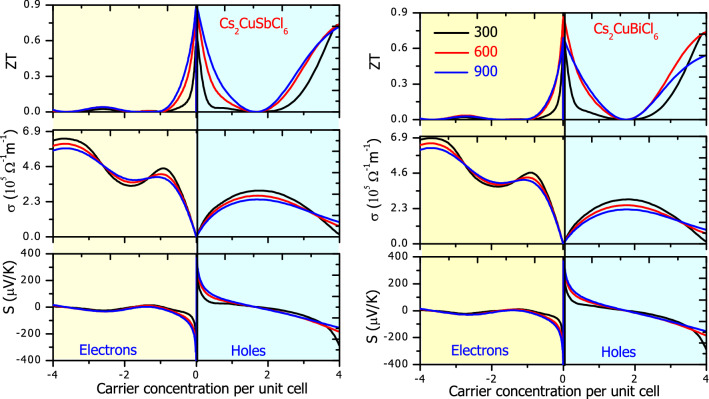
Variation in Seebeck coefficient, conductivity and ZT with electron and hole concentration. Negative carrier concentration is meant for electrons and positive for holes. Different colors are used to distinguish the temperature: Black-300 K; Red-600 K; and Blue-900 K.

Besides band gap, carrier effective mass and carrier concentration are other key parameters for the semiconductor transport performance. The magnitude of the Seebeck coefficient is directly related to the dispersion of energy levels near the Fermi level. The relation of the Seebeck coefficient with carrier concentration and effective mass is: $S=\frac{8{\pi }^{2}{\mathrm{k}}_{\mathrm{B}}^{2}}{3e{\hslash }^{2}}{m}^{\mathrm{*}}T{\left(\frac{\pi }{3n}\right)}^{2/3}$^64^. The relation makes it clear that Seebeck coefficient is directly proportional to effective mass m*, while the magnitude of ‘S’ decreases with an increase in ‘n’. At absolute temperature, semiconductors don’t have free carriers for conduction. Charge carriers are produced only when electrons in the valence band are provided sufficient energy to jump to the conduction band. The VBM and CBM curvatures along with E-K dispersions define the hole and electron effective masses, respectively. Holes have a negative effective mass in comparison to a positive effective mass of electrons. Smaller effective masses mean greater CBM and VBM hybridization. E-K dispersion curves with parabolic curvature have low effective mass, signifies high mobility thereby affording larger electrical conductivity. While high effective mass is the character of the flat band. The energy band in the vicinity of the Fermi level of the entitled materials are less dispersive signify the presence of highly effective mass, which could account high Seebeck coefficient. The various parameters are determined with the help of transM code^69^. The comparative variation in σ, S altogether with inverse effective mass within the vicinity of the Fermi level is presented in Fig. S7. The large Seebeck coefficient around the Fermi level high is due to significant effective mass. As we move away from the Fermi level, the effective mass decreases gradually result in a decrease in the Seebeck coefficient, correspondingly the conductivity increases. The variation in the Seebeck coefficient, the electrical conductivity with carrier doping concentration is presented in Fig. 6. In a semiconductor, the doping of either type of carries amplifies the electrical conductivity. But the Seebeck coefficient being proportional to n^−2/3^ decreases as the doping carrier concentration increases. While the conductivity increases up to a certain value beyond which it decreases. The conductivity increases manifolds with electron doping in comparison to hole doping, it may be because of the high mobility of electrons. The ZT plot conveys optimal hole doping can significantly improve the thermoelectric efficiency.

### Optical properties

The optical properties of a material are directly linked to the dielectric function of the material. These properties are being determined by investigating their visible light energy harvest. This is normally done by calculating the bandgap and the absorption coefficients^70^. Ideally, direct low band gap semiconductor materials possess promising optoelectronic applications like photo-absorbers for solar-cell^71^. The optical properties of a material depend on frequency and they are interconnected with each other if we are capable of calculating one e.g., dielectric function we can extort all other properties easily. The optical properties such as the absorption coefficient, refractive index n (ω), reflectivity R (ω) and conductivity function σ (ω) are obtained from the expression of the real part ε (ω) of the dielectric function^72^.

**Figure 7:**
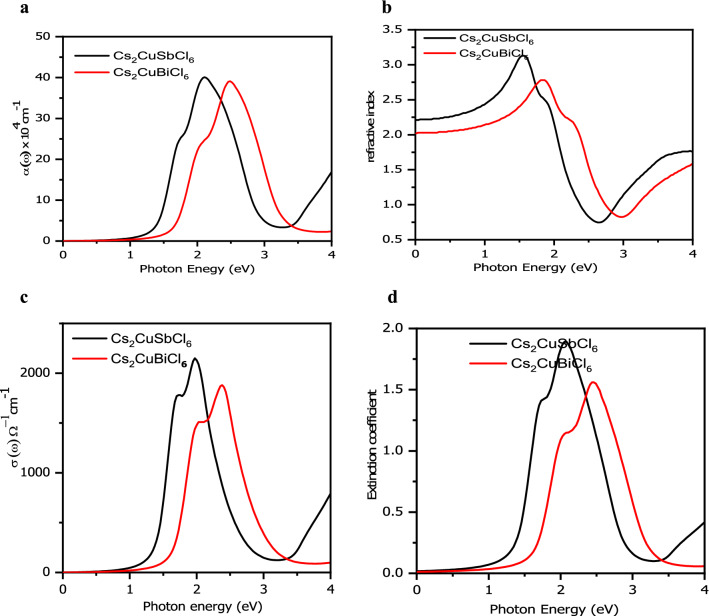
The various optical parameters calculated by GGA + mBJ approximation for Cs_2_CuSbCl_6_ and Cs_2_CuBiCl_6_ where (**a**) represents optical absorption, (**b**) represents optical conductivity, (**c**) represents refractive index, (**d**) represents extinction coefficient.

First, we started with plot of the optical absorption coefficient with photon energy which gives information on the light harvesting capacity of the material. Since, we know that band gap depends inversely upon absorption threshold so materials with higher band gap have narrow absorption in the visible region of electromagnetic spectrum. But the studied materials have smaller band gap showing higher absorption in the visible range as shown in Fig. 7a. These materials show high absorption coefficient ranging from infrared to ultraviolet region and contains entire visible wavelength range. As the photon energy increases the absorption spectrum increases gradually and highest peak occurs at 6.5 eV which corresponds to maximum absorption. This spectrum appeared as a result of electrons exciting from valence to conduction band. The first peak in absorption spectrum for Cs_2_CuSbCl_6_ (Cs_2_CuBiCl_6_) is about 2 eV (2.5 eV) which arises due to transitions from Cu-*d* to M-*p* (M = Sb, Bi) while as second peak occurs at 6 eV (6.5 eV) corresponds to transitions from Cu-*d* to Cl-*p*. The late absorption onset was attributed to the indirect band gap. Further, we tried to investigate the optical conductivity of the materials and plotted in Fig. 7b. It follows a similar trend as the absorption spectra as shown in Fig. 7a. Over the entire photo energy range (0–4 eV) conductivity displays high and low peaks with the presence of hump at particular energies. The maximum conductivity is shown at higher energy range. These calculated curves reveal the same trend or same features as observed in case of reported cases^14,15^. The almost similar nature of band structure leads to similar structure of the optical spectra originates from the top of valence band to the bottom of conduction band. The optical properties determined by the GGA + mBJ + SOC are presented in the supplementary information as displayed in Fig. S8.

An important physical property in optics which provides information about behavior of light inside a material is refractive index. When light is passed through different media, its velocity changes resulting in the variation of refractive of a material. Other important physical quantity which is connected to the light absorption capacity of a material at a particular frequency is called as extinction coefficient. The extinction coefficient is basically complex part of refractive index and represent how electromagnetic wave can propagate in any medium. The variation of refractive index with photon energy is illustrated in Fig. 7c. The static values of refractive index are 2.3 and 2.0 for Cs_2_CuSbCl_6_ and Cs_2_CuBiCl_6_ respectively. They correspond to the values that can be derived from real part of dielectric function. These obtain a maximum value at around 2 eV. From Fig. 7d we see that the extinction coefficient can be divided into three main absorption peaks which are centered on different photon energy range. These various peaks arise because of electronic transitions from one level to another. These all together properties coveys that these inorganic halide double perovskites would be a potential lead-free alternative for optoelectronic device fabrication.

**Figure 8:**
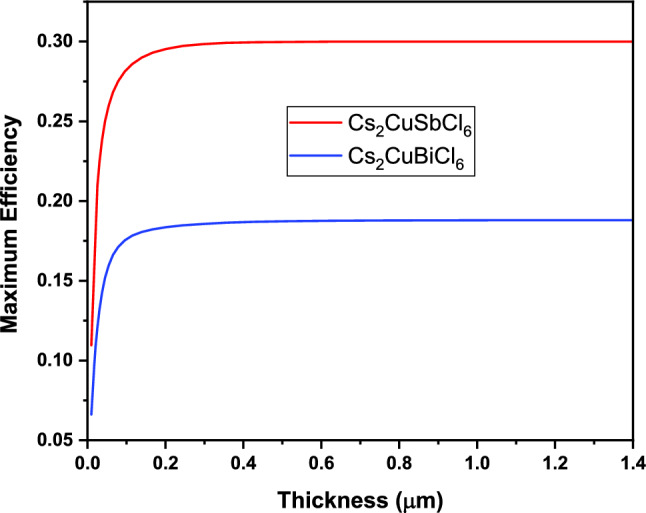
Variation of spectroscopic limited maximum efficiency (SLME) as a function of thickness of absorbing layer.

Spectroscopic limited maximum efficiency: the maximum possible efficiency of a solar absorbing material in PV’s is determined theoretically by Shockley-Queisser limit (SQ) which gives a direct relation between band gap of a material and its maximum power efficiency. The spectroscopic limited maximum efficiency (SLME) is the recently introduced technique which goes beyond the SQ limit to calculate the maximum efficiency of a photovoltaic material by taking into account the absorption coefficient and thickness of material. The SLME takes into account the absorption coefficient as well as radiative/non-radiative recombination losses considering both direct as well as indirect band gap which plays an important role in designing highly efficient photovoltaic device as compared to SQ limit^73,74^. This approach is based on Fermi golden rule. Considering this approach, we have calculated the effect on efficiency of these materials as a function of the thickness of the absorber layers as shown in Fig. 8. Here, we varied the thickness of material from 0 to 1.4 µm and calculated the efficiency of material. Since we know that Cs_2_CuSbCl_6_ perovskite has narrow band gap and high absorption coefficient as compared to Cs_2_CuBiCl_6_ perovskite therefore attains higher power conversion efficiency of about 30% while as Cs_2_CuBiCl_6_ has efficiency about 19%.

In order to inter-relate the dependency of optical and thermoelectric properties on the band structure, we have comparatively plotted the figure of merit, optical absorbance and band structures altogether, referred in the Fig. S9. The narrow band gap Sb-based perovskite shows more absorption peaks in the visible region compared to Bi-based perovskite, due to its small bandgap. On comparing the magnitude of ZT, low band gap Cs_2_CuSbCl_6_ material also have slightly higher ZT-peaks in vicinity of Fermi level in comparison to Cs_2_CuBiCl_6_ perovskite. Although Cs_2_CuBiCl_6_ show high Seebeck coefficient but still it shows lower ZT peaks. It is because electrical conductivity an important factor for ZT is low for wide bandgap materials. So, narrow band gap materials are most suitable for both applications. If the band gap is reduced to zero, the conductivity may get amplified but Seebeck decreases drastically, so the ZT gets dejected.

## Conclusion

In the present study the structural stability along with electronic, elastic, thermoelectric and optical properties of inorganic halide double perovskites have been calculated. Both the materials are stable in cubic structure follow the space symmetry of the Fm-3m space group. The stability in the Fm-3m space group is defined with the help of energy optimization, tolerance factor. Moreover, the positive values of elastic constants authenticate the mechanical stability of the materials. The elastic constants further confirm the ductile and anisotropic nature of the materials. The band structure and density of states reflect the semiconducting character with small indirect ban gap. These materials have excellent optical absorption in the visible range can be used for optoelectronic application purpose. The high Seebeck coefficient with low thermal conductivity is responsible for high figure of merit close to unity.

## Supplementary Information

Supplementary Information.

## Data Availability

The data would be available from the corresponding author on reasonable request.
