# Supplementary material for: Potential lead-free small band gap halide double perovskites Cs2CuMCl6 (M = Sb, Bi) for green technology
Source: Sci Rep. 2021 Jun 21;11:12945. doi: 10.1038/s41598-021-92443-1 (PMC8217524; doi:10.1038/s41598-021-92443-1)
Supplement: Supplementary file 1 — Supplementary Information. [file 41598_2021_92443_MOESM1_ESM.pdf]

Supplementary Information

Potential Lead-free small band gap halide double perovskites Cs_2CuMCl_6 (M=Sb, Bi) for green energy technology

Muskan Nabi and Dinesh C. Gupta[#]
Condensed Matter Theory Group, School of Studies in Physics
Jiwaji University, Gwalior – 474 011 (INDIA)
Corresponding Author e-mail: sosfizix@gmail.com[#]

Structural properties

Periodic Table of Elements

1	2	3	4	5	6	7	8	9	10	11	12	13	14	15	16	17	18
H																	He
Li	Be											B	C	N	O	F	Ne
Na	Mg											Al	Si	P	S	Cl	Ar
K	Ca	Sc	Ti	V	Cr	Mn	Fe	Co	Ni	Cu	Zn	Ga	Ge	As	Se	Br	Kr
Rb	Sr	Y	Zr	Nb	Mo	Tc	Ru	Rh	Pd	Ag	Cd	In	Sn	Sb	Te	I	Xe
Cs	Ba	*	Hf	Ta	W	Re	Os	Ir	Pt	Au	Hg	Tl	Pb	Bi	Po	At	Rn
Fr	Ra	**	Rf	Db	Sg	Bh	Hs	Mt	Ds	Rg	Cn	Nh	Fl	Mc	Lv	Ts	Og
		*	La	Ce	Pr	Nd	Pm	Sm	Eu	Gd	Tb	Dy	Ho	Er	Tm	Yb	Lu
		**	Ac	Th	Pa	U	Np	Pu	Am	Cm	Bk	Cf	Es	Fm	Md	No	Lr

- alkaline-earth metal halide perovskites
- metal chalcogenide perovskite
- transition metal halide perovskites
- Group-14 element halide perovskites
- heterovalent metal halide perovskites
- lanthanide and actinide halide perovskites

Fig. S1(a): The substitution of lead byhomo-valent elements in perovskite type materials from the periodic table of elements to form stable lead-free materials for various applications.

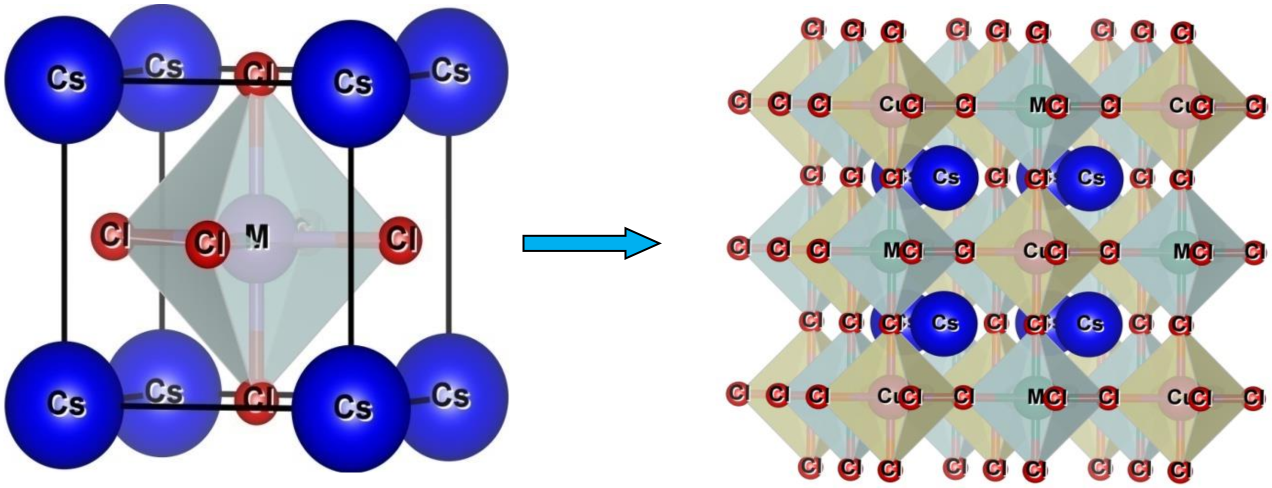

Fig. S1 (b): Pictorial representation of Cs_2CuMCl_6 (M = Sb, Bi) perovskite in which cesium is enclosed by a cage of 12 halide atoms (Cl) while as *d* block element Cu and *p* group elements (Sb/Bi) lie in octahedral of halide atoms having coordination 6 to these atoms. The structural analysis is being carried out by using VESTA software [https://doi.org/10.1107/S0021889808012016]

Second-order elastic constants and Mechanical Stability

Table S1: Maximum and minimum values of Young's modulus, linear compressibility, shear modulus and Poisson's ratio.

Material	Young's modulus		Linear compressibility		Shear modulus		Poisson's ratio	
	Y_{min}	Y_{max}	β_{min}	β_{max}	B_{min}	B_{max}	ν_{min}	ν_{max}
$Cs_2CuSbCl_6$	14.39	47.24	10.38	10.38	5.05	18.82	0.09	0.72
$Cs_2CuBiCl_6$	11.65	46.17	10.48	10.48	4.05	18.35	0.80	0.76

Table S2: Average values of different elastic moduli calculated by the Reuss-Vogit-Hill scheme.

Material	B			G			Y			ν		
	B_v	B_R	B_H	G_v	G_R	G_H	Y_v	Y_R	Y_H	V_V	V_R	V_H
$Cs_2CuSbCl_6$	32.11	32.11	32.11	10.56	7.13	8.84	28.55	19.94	24.31	0.35	0.39	0.37
$Cs_2CuBiCl_6$	31.79	31.79	31.79	9.77	5.88	7.82	26.59	16.62	21.70	0.36	0.41	0.38

Table S3: Pure longitudinal (V_L) and two transverse (V_{T_1} andV_{T_2}) velocities along [100], [110] and [111] direction.

**Material**	**V_L**			**V_{T1}**			**V_{T2}**		
	**[100]**	**[110]**	**[111]**	**[100]**	**[110]**	**[111]**	**[100]**	**[110]**	**[111]**
**$Cs_2CuSbCl_6$**	3880	3380	3200	1150	1150	1930	1150	2230	1930
**$Cs_2CuBiCl_6$**	3620	3120	2940	960	960	1780	960	2080	1780

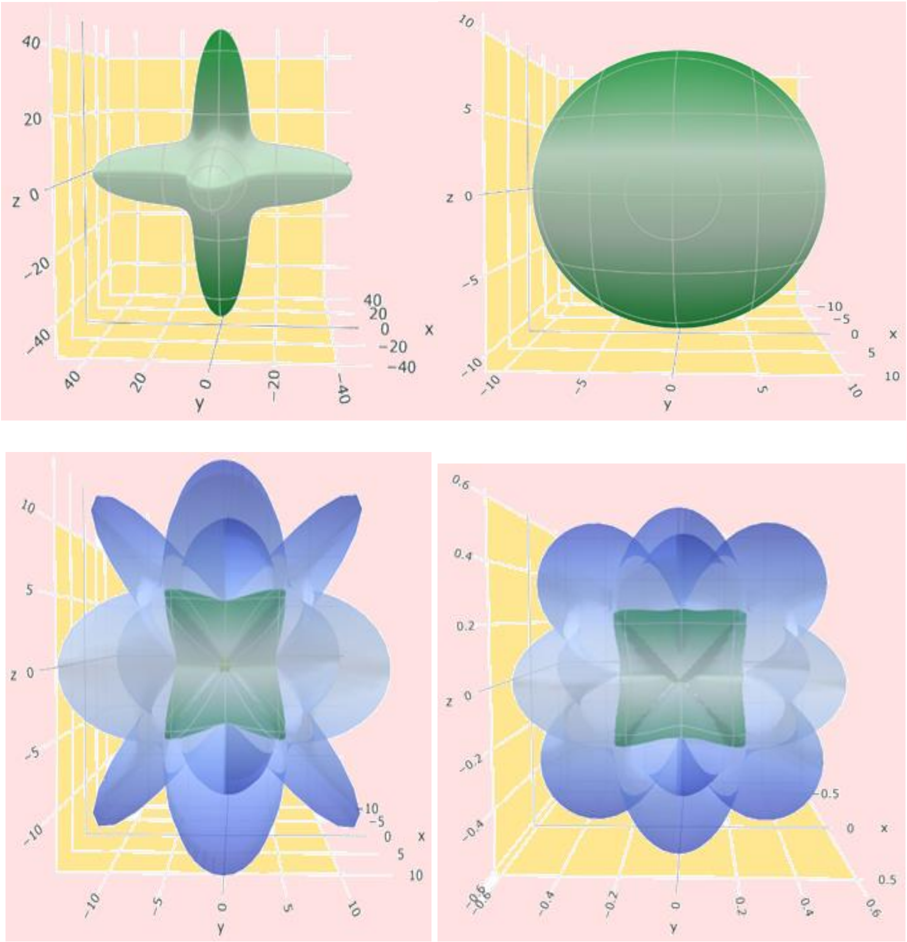

Figure S2(a): 3-D representation of Young's modulus, linear compressibility, Shear modulus and Poisson's ratio for $Cs_2CuSbCl_6$

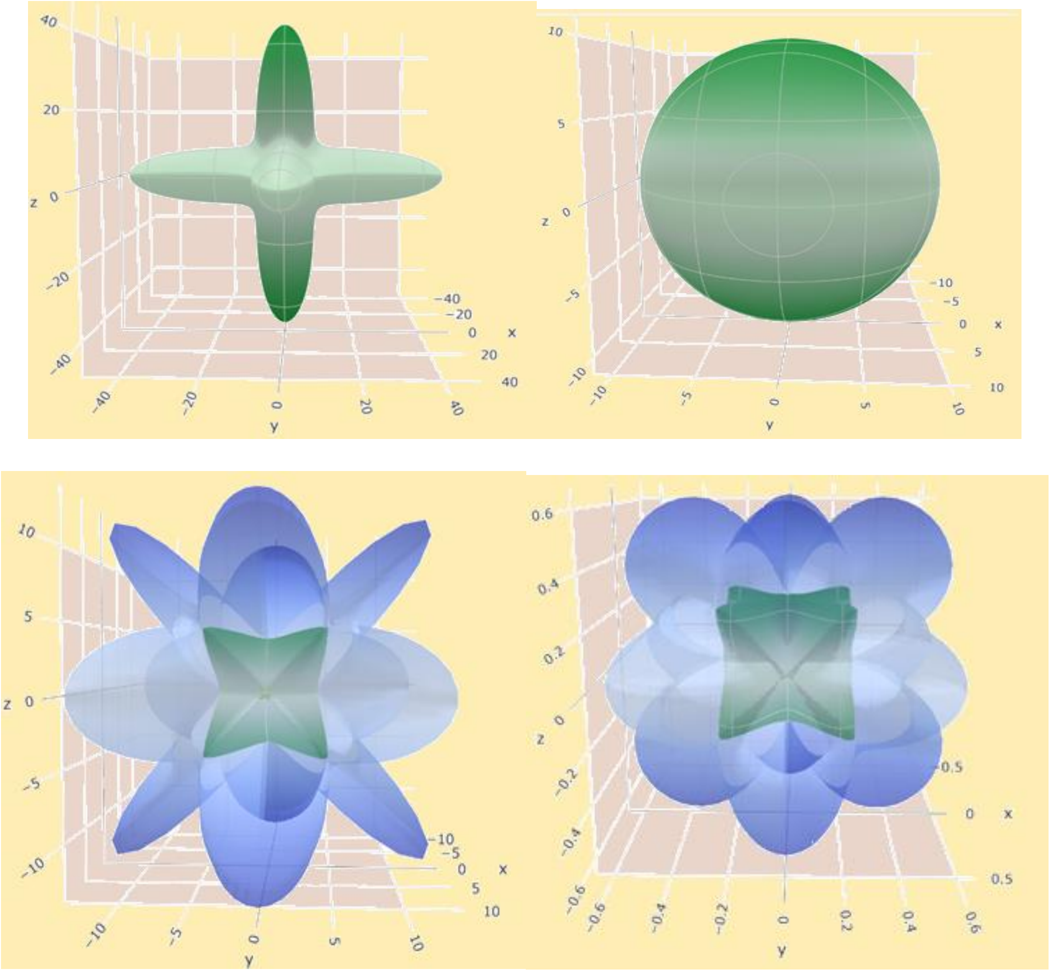

Figure S2(b): 3-D representation of Young's modulus, linear compressibility, Shear modulus and Poisson's ratio for $Cs_2CuBiCl_6$

Electronic properties:

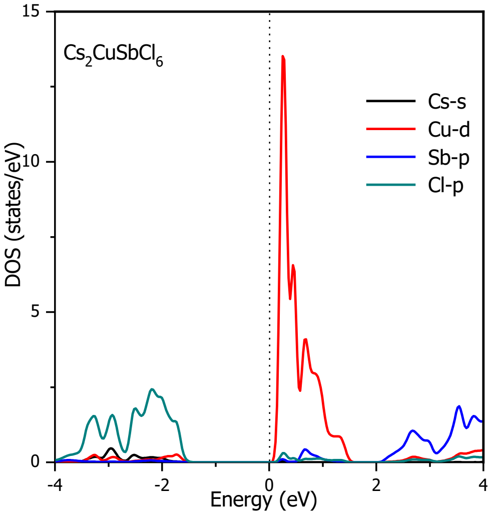

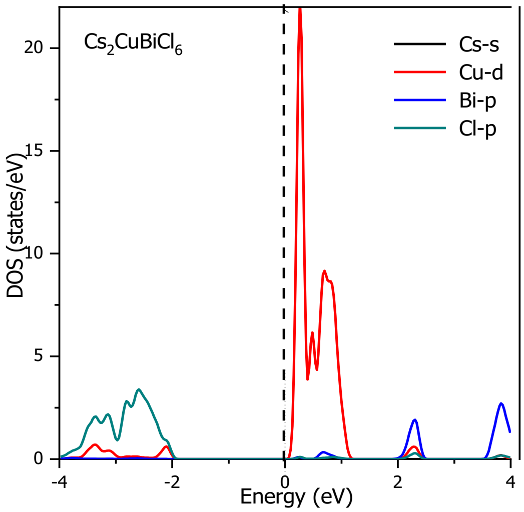

Fig. S3: The projected density of states (pDOS) provides insights to quantitative and qualitative distribution of energy states. The energy states in the vicinity of the Fermi level predominantly control the electronic properties. The pDOS obtained by the GGA+mBJ+SOC are plotted in energy range of -4 eV to 4 eV as shown above**.** The energy states of Cs are nowhere in the neighbourhood of Fermi level, either located deep down in valence band or at higher energies above Fermi level. The DOS peaks around -4.0 eV to -2.0 eV in both materials are contributed mostly Cl-atom p-states with very small contribution Cu *d*-states. The *d*-states of Cu form the valence band minima. The Bi-*p* states form the sub-bands in the conduction band above the d-states of Cu. The splitting of the p-states of M-atom by the inclusion of SOC effect.

Transport properties:

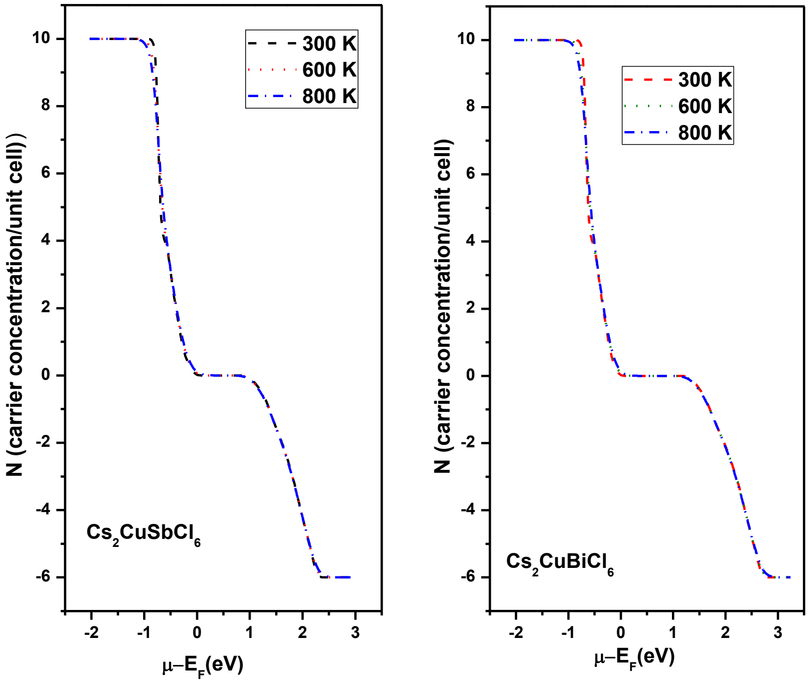

Fig. S4: Variation of carrier concentration with chemical potential at different temperatures for $Cs_2CuSbCl_6$ and $Cs_2CuBiCl_6$

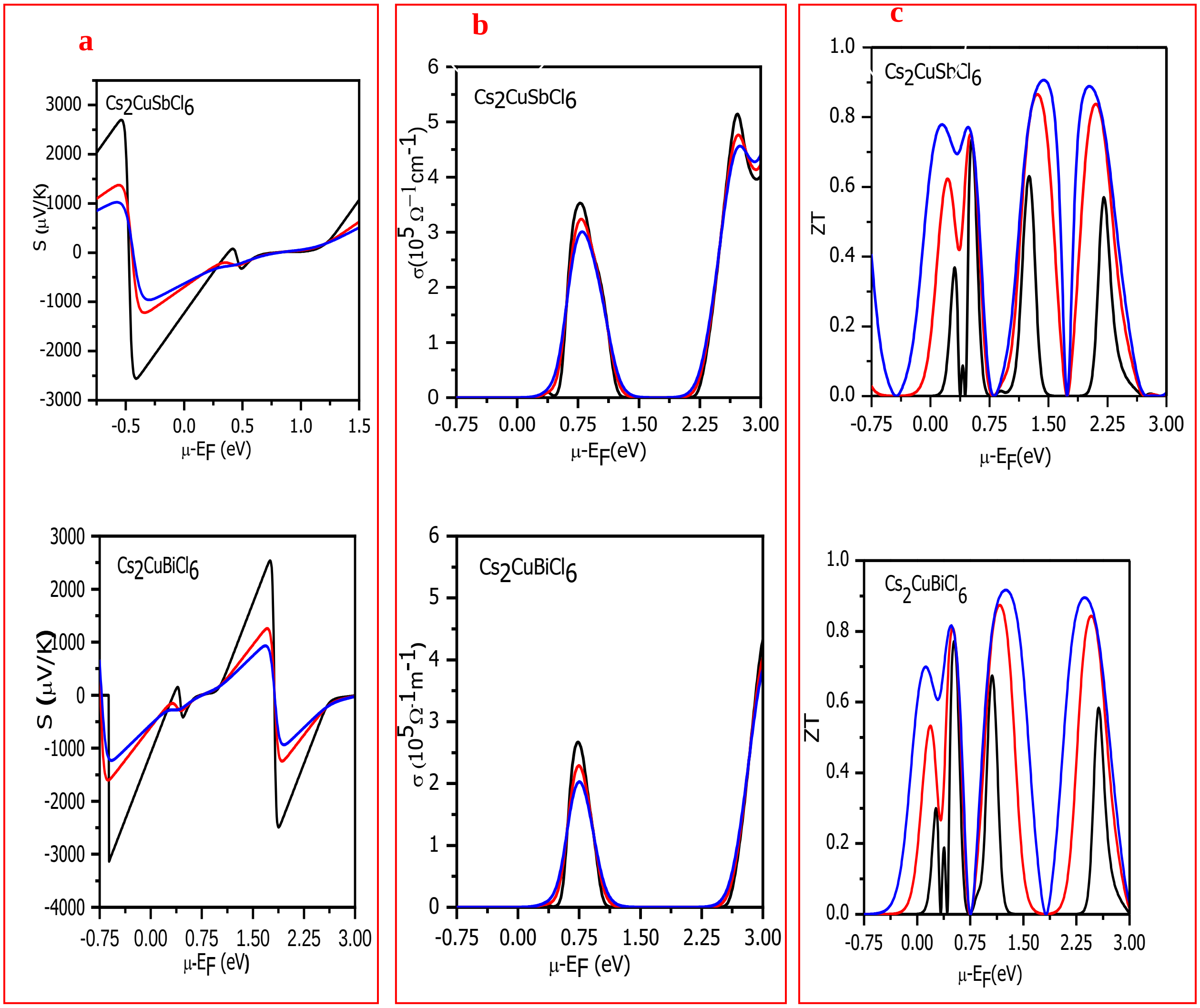

Fig. S5: The variation in transport properties namely (a) Seebeck Coefficient; (b) Electrical conductivity; (c) Figure of merit (ZT) obtained by GGA+mBJ+SOC method. The variation observed is similar to that obtained by GGA+mBJ method. However, peaks get shifted and peak values are altered, it is due to change in the bandgap and shifting of energy states with incorporation of SOC. The zero conductivity signifies the bandgap/pseudo-gap corresponding to which Seebeck coefficient possess a peak. The ZT exhibits the major peaks for positive chemical potential indicate hole doping could improve the thermoelectric efficiency of the materials.

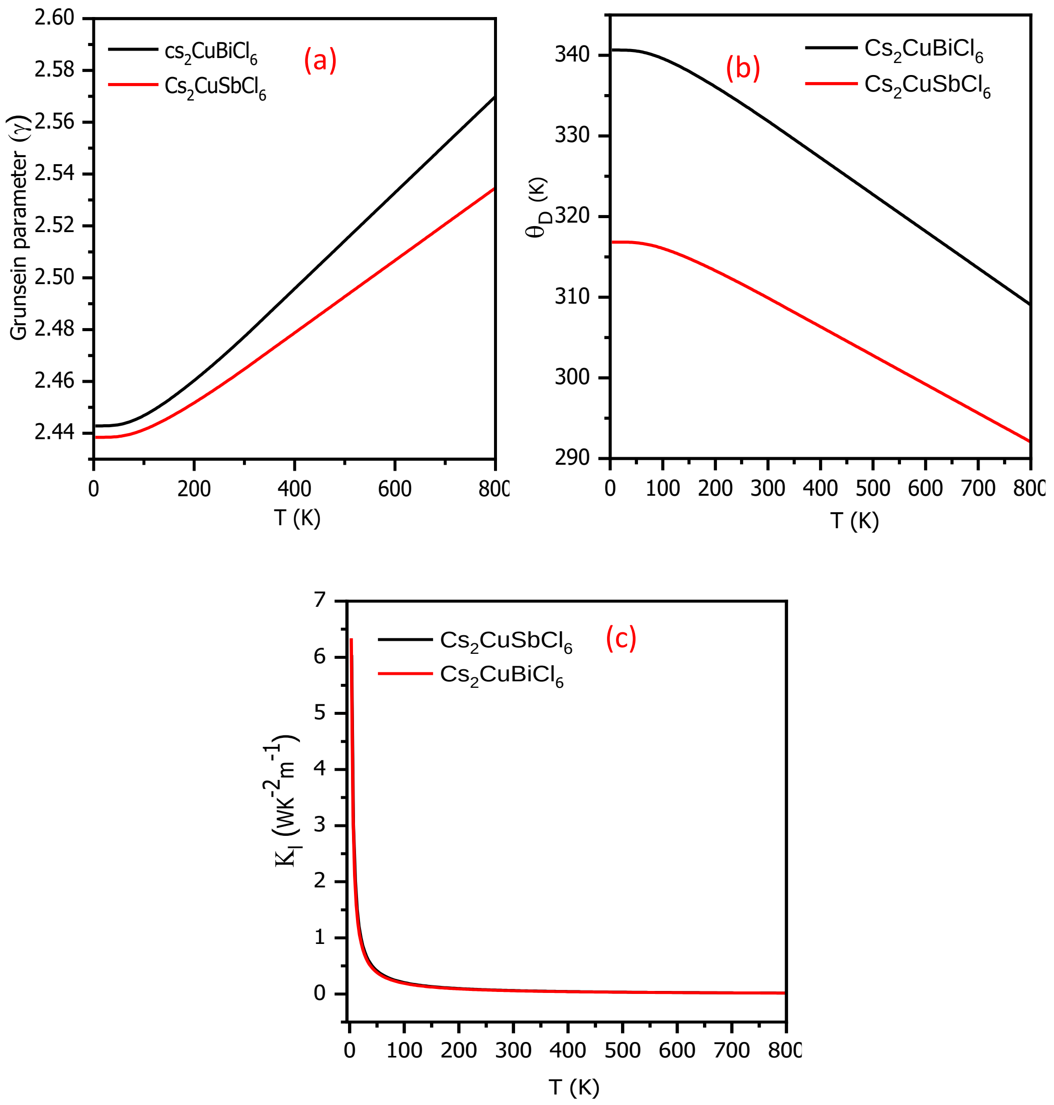

Fig. S6: Variation in different physical parameters with temperature for Cs_2CuMCl_6 (M=Sb, Bi) perovskites: (a) Grüneisen parameter; (b) Debye temperature; (c) lattice thermal conductivity.

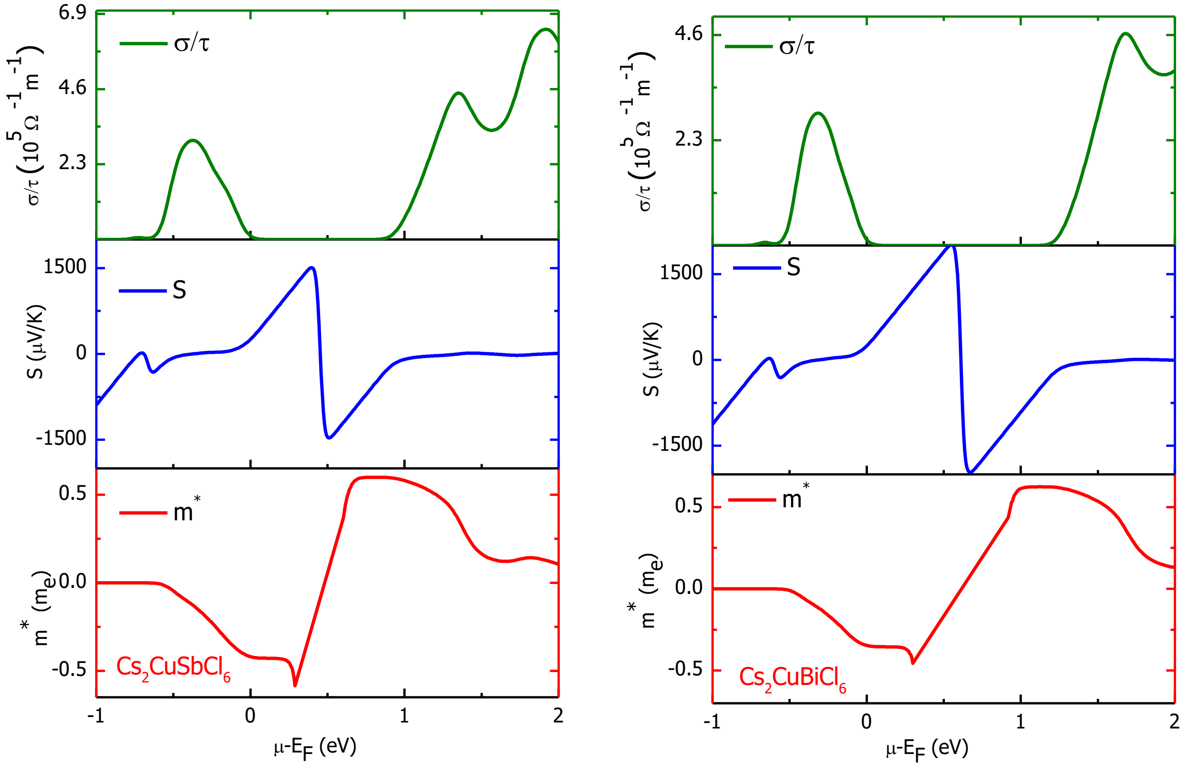

Fig. S7: The comparative variation in σ, S and inverse effective mass within the vicinity of the Fermi level with chemical potential.

Table S4: Obtained ZT by GGA+mBJ method for Cs_2CuMCl_6double perovskites in comparison with earlier reported results in the literature

Material	Highest ZT	DOI
**$Cs_2CuSbCl_6$**	0.92	PW
**$Cs_2CuBiCl_6$**	0.90	PW
**$CsSnI_{3-x}Cl_x$**	0.14	10.1038/s41467-019-13773-3
**Cs_2PtI_6**	2.4	10.1021/acsaem.0c02236
**$Cs_2BiAgCl_6$**	0.78	arXiv:1801.03703
**$Cs_2BiAgBr_6$**	0.78	arXiv:1801.03703
**Cs2InAgCl$_6$**	0.94	10.1016/j.cocom.2019.e00374
**$Cs_2NaTiCl_6$**	0.90	10.1016/j.jallcom.2020.156000
**Cs_2NaVCl_6**	0.90	10.1016/j.jallcom.2020.156000
Abbreviation: PW-present work		

Optical properties:

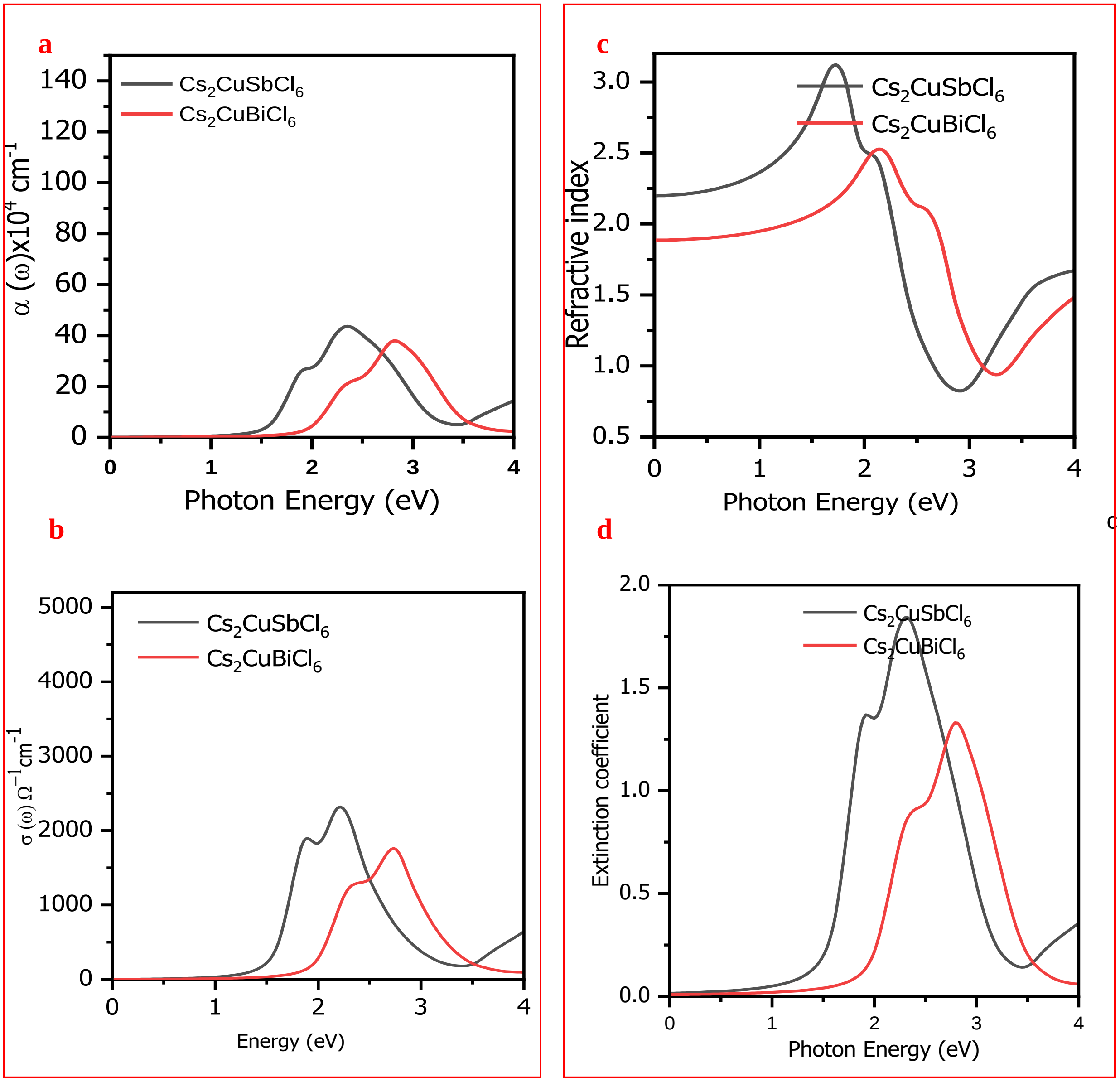

Fig S8: The optical parameters like optical absorption 7 (a), optical conductivity 7(b), refractive index 7 (c), extinction coefficient 7(d) calculated by GGA+mBJ+SOC approximation for $Cs_2CuSbCl_6$ and $Cs_2CuBiCl_6$.

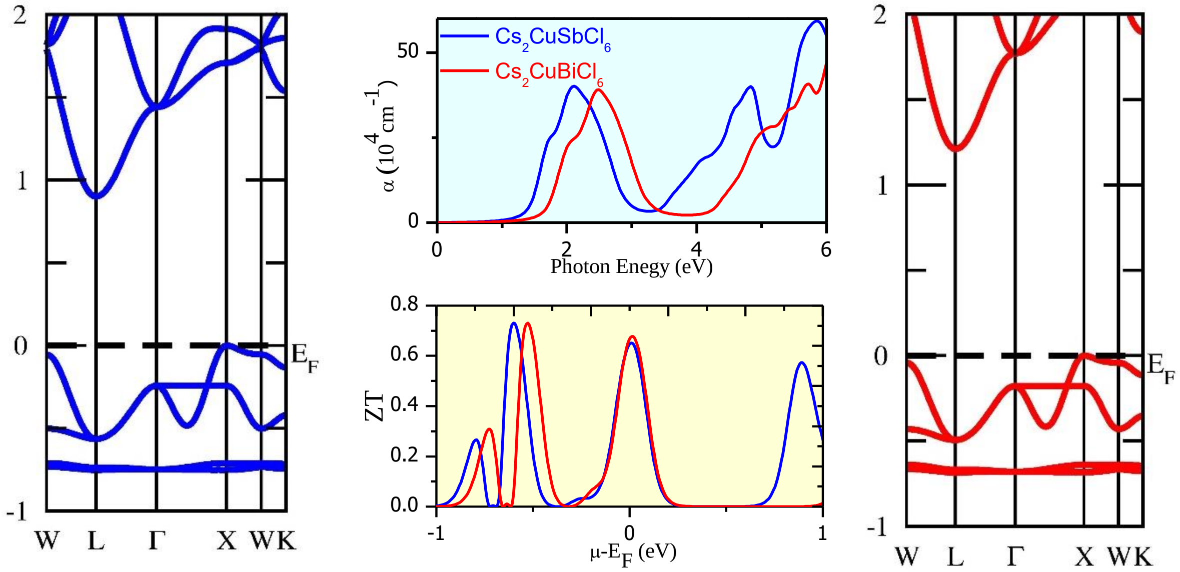

Fig S9: Variation in the ZT and absorption coefficient of Cs_2CuMCl_6 halide perovskites altogether with the band profile. Left side bad structure id of $Cs_2CuSbCl_6$ and right extreme is of $Cs_2CuBiCl_6$. The blue color lines are used for $Cs_2CuSbCl_6$ and red color is used for $Cs_2CuBiCl_6$.
